# Dual BAFF/APRIL inhibition with telitacicept in systemic lupus erythematosus and IgA nephropathy: pharmacological rationale, clinical efficacy, and safety on female fertility preservation

**DOI:** 10.3389/fphar.2026.1794203

**Published:** 2026-05-20

**Authors:** Hongzhu Liu, Hongyu Yu, Jia Lü, Haoyue Tang, Xuehong Lu

**Affiliations:** 1 Department of Nephrology, The Second Hospital of Jilin University, Changchun, China; 2 Department of Nephrology, Jilin City People’s Hospital, Jilin City, Jilin, China

**Keywords:** BAFF/APRIL inhibition, female fertility preservation, IgA nephropathy, SLE, telitacicept

## Abstract

**Background:**

Systemic lupus erythematosus (SLE) and IgA nephropathy (IgAN) are immune-mediated renal disorders characterized by aberrant B-cell activation. While SLE shows a striking female predominance, IgAN is more prevalent in males; nonetheless, both conditions typically peak the during reproductive years. Their pathogenesis involves distinct molecular drivers: SLE is mediated by specific autoantibodies, whereas IgAN is initiated by galactose-deficient IgA1 (Gd-IgA1) acting as a primary antigen that elicits immune complex deposition. Conventional immunosuppressive therapies are effective but limited by non-selective toxicity, particularly in women of reproductive age. Telitacicept, a first-in-class recombinant transmembrane activator and CAML interactor (TACI)–fragment crystallizable (Fc) fusion protein, simultaneously neutralizes B-cell activating factor (BAFF) and a proliferation-inducing ligand (APRIL), offering a targeted pharmacological strategy.

**Methods:**

This narrative review synthesizes evidence from randomized controlled trials, real-world studies, and mechanistic investigations to evaluate the pharmacological rationale, clinical efficacy, and safety profile of telitacicept in SLE and IgAN, with particular attention to its steroid-sparing effects and endocrine safety.

**Results:**

Dual inhibition of BAFF and APRIL by telitacicept suppresses pathological B-cell maturation and plasma cell survival, leading to marked reductions in antigen Gd-IgA1 and associated immune complexes, proteinuria, and disease activity, alongside stabilization of renal function. Clinical trials and real-world data demonstrate robust efficacy in both refractory IgAN and active SLE/lupus nephritis, with sustained complement recovery and favorable tolerability. Importantly, available clinical data indicate no direct gonadotoxic effects, with preserved ovarian reserve markers and hypothalamic–pituitary–gonadal axis function, distinguishing telitacicept from cytotoxic immunosuppressants.

**Conclusion:**

Telitacicept represents a pharmacologically rational, targeted immunomodulator that combines effective disease control with a favorable safety profile. Its dual BAFF/APRIL inhibition and steroid-sparing properties support its potential role as a long-term therapeutic option for patients with SLE and IgAN, particularly women requiring sustained disease control who seek to minimize systemic toxicity and safeguard ovarian reserve.

## 1 Introduction

SLE and IgAN, among the most common immune-mediated renal diseases encountered in clinical practice, are characterized by chronic progression and frequently involve the kidneys early in the disease course. SLE exhibits a marked female predominance, accounting for approximately 90% of all reported cases, most of whom are diagnosed during the reproductive age range of 15–44 years. As opposed to SLE, IgAN shows a male predilection; however, its clinical onset frequently peaks between ages 20 and 40. This demographic overlap places many female patients at risk during their most active reproductive years (Al-Riyami et al., 2021; Weng et al., 2025). Pregnant women diagnosed with SLE face a substantially higher vulnerability to adverse pregnancy outcomes (APOs) than the general population, comprising preterm labor, miscarriage, fetal growth restriction, and preeclampsia, which occur more frequently than in the healthy population. Such epidemiological patterns highlight the intrinsic link between clinical management and long-term female fertility preservation in SLE and IgAN, underscoring the need to meticulously coordinate pregnancy timing with disease remission (Braga et al., 2021; Jarrick et al., 2021). Yet, the gonadal impairment linked to traditional immunosuppressive agents creates substantial hurdles in the medical management of women in reproductive prime. As a standard treatment for lupus nephritis (LN) induction, cyclophosphamide (CYC) is linked to documented, dose-related toxicity toward the ovaries, which can culminate in a lasting loss of ovarian function (Palmer et al., 2017; Chehab et al., 2019; Sharma et al., 2020). Despite its superior profile regarding gonadal toxicity compared to CYC, mycophenolate mofetil (MMF) is prohibited for pregnant or soon-to-be pregnant women owing to its adverse effects on the embryo (Palmer et al., 2017).

Furthermore, prolonged or intensive glucocorticoid treatment may impair regular menstruation and ovulatory function by inhibiting the hypothalamic–pituitary–gonadal (HPG) axis (Khizroeva et al., 2019). Overall, these drawbacks necessitate innovative treatments that are better tolerated and more specific to support the enduring health of women during their reproductive prime. Telitacicept is a novel biologic agent that simultaneously blocks BAFF and APRIL, thereby inhibiting the production of Gd-IgA1 (antigen) and pathogenic autoantibodies, selectively inhibiting aberrant B-cell activation and immune complex formation. Telitacicept achieves substantial control over disease symptoms, effectively lowering the dosage of glucocorticoids and toxic immunosuppressants that pose risks to fertility (Liu et al., 2025). Therefore, by achieving clinical remission with superior tolerability, telitacicept provides a novel pathway to safeguard ovarian function, enhance pre-gestational health, and mitigate the risk of pregnancy complications in patients with immune-related nephropathies (Hu et al., 2025).

## 2 Epidemiological characteristics, fertility preservation, and pregnancy challenges in SLE and Iga nephropathy

### 2.1 Epidemiological characteristics

SLE is a systemic autoimmune disease characterized by substantial regional and demographic heterogeneity. Although its overall prevalence is relatively low, the disease burden and long-term prognostic risks are considerable. Global SLE incidence and prevalence exhibit significant regional and ethnic disparities, peaking in North America (23.2 and 241 per 100,000 person-years/people, respectively) with a female-to-male ratio of approximately 6:1 (Rees et al., 2017).

With women representing roughly 90% of all reported diagnoses, SLE shows a clear female predominance, primarily affecting individuals during their peak reproductive window between the ages of 15 and 44 years. These demographic patterns underscore the need for therapeutic approaches that balance protection of reproductive potential with sustained immune regulation (Gholizadeh Ghozloujeh et al., 2024). With a yearly occurrence of roughly 2.5 per 100,000, IgAN remains the leading primary glomerular condition worldwide, though its frequency among biopsy-verified renal cases varies significantly across different geographical areas. In Europe and North America, IgAN accounts for roughly 10%–20% of all reported cases, whereas it constitutes a much larger share in East Asia, likely due to divergent genetic backgrounds and various environmental factors (McGrogan et al., 2011). While IgAN is more prevalent in males, its clinical onset typically occurs between the ages of 20 and 40 years. This demographic profile encompasses a substantial number of women during their vital reproductive years. Accordingly, therapeutic strategies for IgAN must integrate family planning, safeguarding reproductive endocrine function, and a thorough analysis of the toxic risks posed by medications (Şener and Şener, 2025).

### 2.2 Impact of disease activity on female fertility and pregnancy outcomes

#### 2.2.1 Central role of preconception disease control in pregnancy outcomes

Current clinical guidelines and expert consensus (Gholizadeh Ghozloujeh et al., 2024) recommend that women of reproductive age with immune-mediated diseases maintain low disease activity or achieve clinical remission for at least 6 months—ideally 12 months—before attempting conception. This approach intends to mitigate negative pregnancy consequences and forestall permanent organ injury in the mother triggered by active immune-driven inflammation while pregnant (Gholizadeh Ghozloujeh et al., 2024). Extensive pooled analyses of cohort data highlight that pre-pregnancy disease regulation is the most critical factor influencing the eventual outcomes of pregnancy in these patients. Among the most robust predictors of APOs, pre-existing renal impairment, reflected by reduced estimated glomerular filtration rate (eGFR), and persistent proteinuria—commonly defined as >1.0 g/day—have been repeatedly validated as independent risk factors. For IgAN patients, achieving urinary protein excretion below 0.5 g/day prior to gravidity—typically considered a vital milestone for ensuring gestational safety—is a key goal (Wang et al., 2019).

#### 2.2.2 Influence of disease activity and immunologic factors on pregnancy risk

Pregnancy outcomes in women with SLE are shaped by a complex interplay of immunologic and clinical factors. Pathological activity, LN, antiphospholipid syndrome (APS) or antiphospholipid antibodies (aPL) presence, and pregnancy-induced hypertension are distinct and major factors that forecast adverse pregnancy outcomes (Ku et al., 2016; Sun et al., 2025).

Thus, the core therapeutic priorities for improving gestational outcomes involve achieving remission before pregnancy and sustaining that stability throughout the gestational period to protect both patients (Sun et al., 2025). Uncontrolled SLE, especially when LN manifests during gestation, poses a major threat to pregnancy, significantly elevating the incidence of fetal loss, spontaneous abortion, premature delivery, and preeclampsia (Ku et al., 2016). Clinical flares, depleted complement components, and aPL-positive status are potentially potent contributors to a higher incidence of adverse pregnancy outcomes (Braga et al., 2021; Le Guern et al., 2024; Kong et al., 2025). A recent meta-analysis by Wind et al. (2024) corroborated these findings, identifying aPL positivity, active SLE in early pregnancy, and reduced complement levels as significant predictors of APOs risk. Similarly, patients with IgAN constitute an intrinsically high-risk pregnancy population. A matched cohort study by Liu et al. (2014) reported significantly increased rates of APOs among pregnant women with IgAN, including severe preeclampsia, intrauterine stillbirth, fetal injury, fetal malformation, and miscarriage, highlighting the substantial pregnancy-related risk burden in this group. Regarding SLE, prospective data indicate that premature delivery rates may reach 49% in active LN cases, yet these obstetric outcomes are significantly improved if clinical stability is established prior to pregnancy (Moroni et al., 2016). While clinical activity is the primary adjustable risk factor, the application of traditional immunosuppressants, such as MMF and CYC, is limited due to their adverse effects on female fertility and pregnancy safety. Such constraints frequently impede the achievement of profound clinical quiescence during the critical window just before fertilization for fertile women (Le Guern et al., 2024).

Against this background, telitacicept potentially excels by utilizing a selective, non-cytolytic therapeutic approach that avoids extensive biological injury to the host. Telitacicept reduces systemic lupus erythematosus disease activity index (SLEDAI) markers, elevates complement proteins, and inhibits immune complex formation. This allows for a reduced intake of high-risk immunosuppressants, thus establishing an ideal physiological window for women planning to conceive (Liu et al., 2014). By targeting the BAFF/APRIL signaling axis to regulate B-cell functions, telitacicept emerges as an encouraging candidate for prolonged maintenance care in female patients within their childbearing years.

## 3 Impact on fertility and pregnancy safety of conventional immunosuppressants: therapeutic dilemmas in women of reproductive age

Despite the essential role of steroids and traditional immune-modulating drugs in stabilizing SLE and IgAN, ensuring long-term safety for female patients’ fertility preservation during their fertile years remains challenging (Barber et al., 2021). By promptly neutralizing immune-mediated inflammatory responses, glucocorticoids remain an essential mainstay in current clinical management; however, prolonged or moderate-to-high dose exposure can exert negative feedback on the HPG axis, leading to impaired gonadotropin secretion, ovulatory dysfunction, menstrual irregularities, and reduced ovarian reserve (Khizroeva et al., 2019; Bouazza et al., 2025). In LN, CYC remains a classic induction therapy, despite its established adverse effects on female fertility preservation.

As evidenced by a pooled analysis, elevated cumulative CYC exposure is a major factor in the development of menstrual cessation (Chen et al., 2017). Due to its properties as an alkylating cytotoxin, CYC causes direct structural injury to the ovarian stroma and exhausts the primordial follicle pool, frequently leading to irreversible premature menopause. The risk of gonadal toxicity is closely related to cumulative dose, patient age, and route of administration; notably, exposure to a combined dose greater than 8 g is associated with a significantly high risk of ovarian failure (Ponticelli and Moroni, 2015). Supporting these findings, Sharma et al. (2020) reported that even moderate-dose CYC therapy was associated with significant reductions in anti-müllerian hormone (AMH) levels, ovarian volume, and antral follicle count. Although MMF exhibits a milder impact on ovarian reserves, its proven teratogenic potential makes it absolutely prohibited during gestation, requiring a mandatory washout period of 6 weeks before a planned pregnancy (Zhang et al., 2020; Gholizadeh Ghozloujeh et al., 2024). In comparison, calcineurin-targeting agents, such as tacrolimus (TAC), are associated with fewer adverse effects on gonadal function and female fertility. Existing data confirm that TAC is generally well tolerated during pregnancy, as it does not seem to increase the risk of gestational complications (Lazzaroni et al., 2016). A 2024 retrospective study by Nakai et al. (2024) demonstrated that the incidence of APOs and adverse maternal outcomes (AMOs) among pregnant patients receiving TAC did not significantly differ from that in non-exposed controls. Nevertheless, calcineurin inhibitor (CNI)-based therapy is not without limitations. The potential for renal toxicity, elevated blood pressure, and impaired glucose metabolism during chronic CNI treatment continues to be a major consideration, especially for LN patients on extended maintenance (Ichinose et al., 2018).

Additionally, TAC’s sensitive dosing requirements demand frequent pharmacological monitoring (maintaining <6.0 ng/mL) to reduce the likelihood of poor intrauterine growth or preterm birth, thereby increasing management pressure on both patients and clinicians (Nakai et al., 2024). Taken together, such restrictions underscore the need for reliable immune-regulating protocols tailored to the unique requirements of fertile women with LN. In this light, telitacicept stands out as a pioneering B-lymphocyte modulator that inhibits both BAFF and APRIL, potentially providing unique benefits for female patients in fertile years (Le Guern et al., 2024). By moderating B-lymphocyte overactivation and preventing immune complex buildup, telitacicept effectively improves SLEDAI markers and complement levels. Crucially, it enables a reduction in high-risk immunosuppressants (e.g., MMF and CYC), thereby optimizing long-term safety and ensuring that patients achieve optimal disease stability before attempting to conceive (Jiang et al., 2023; Dong et al., 2025). Moreover, telitacicept eliminates the need for standardized drug-level tracking. In contrast to the rigorous blood-concentration oversight essential for CNI therapy, its more straightforward dosing regimen likely boosts sustained compliance and practical utility for reproductive-aged women with SLE (Nakai et al., 2024). The safety profiles regarding female fertility preservation and clinical considerations for various therapeutic agents used in SLE and IgAN are presented in Table 1.

**TABLE 1:** Comparison of fertility preservation and pregnancy safety: traditional immunosuppressants vs. telitacicept.

Drug class	Representative agent	Therapeutic role in SLE/IgAN	Major female fertility toxicity and risk	Pregnancy planning considerations	Comparative pharmacological value of telitacicept
Cytotoxic agents	CYC	Severe LN induction	Alkylating cytotoxicity leading to direct ovarian follicle depletion and stromal damage	Generally avoided in childbearing age	Gonadotoxicity-free: Reduces reliance on CYC, preserving ovarian reserve
Purine synthesis inhibitors	MMF	LN induction and maintenance	High risk of fetal malformations and loss	Absolute contraindication during pregnancy; requires ≥6-week washout before conception	Non-teratogenic mechanism: Eliminates MMF-associated developmental risks
Glucocorticoids	Methylprednisolone	Foundational therapy across disease stages	HPG axis suppression; ovulatory and metabolic dysfunction	Dose minimization required during pregnancy	Steroid-sparing: Reduces endocrine disruption and long-term metabolic side effects
Calcineurin inhibitors	TAC	Maintenance therapy	Nephrotoxicity; risk of pregnancy-induced hypertension	Dose minimization required during pregnancy	Simplified management: No nephrotoxicity or need for complex TDM
BAFF/April inhibitors	Telitacicept	Targeted biologic therapy	Protects ovarian reserve AMH and HPG axis without gonadotoxicity	Discontinue prior to conception (data limited)	Integrated efficacy: Combines targeted control with superior potential for female fertility preservation

## 4 Fertility preservation mechanisms and clinical evidence of telitacicept in autoimmune renal diseases

### 4.1 Mechanistic role of telitacicept in the immunopathology of IgA nephropathy and SLE

**FIGURE 1:**
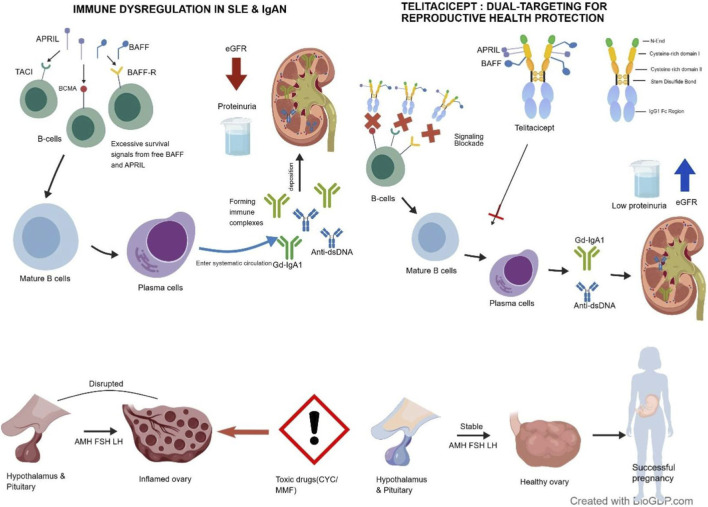
In SLE and IgAN, excessive BAFF and APRIL signaling through BAFF-R, TACI, and BCMA promotes abnormal B-cell survival and differentiation into plasma cells. This leads to increased production of key pathogenic factors, specifically the antigen Gd-IgA1 in IgAN and anti-dsDNA autoantibodies in SLE. These molecules form immune complexes that deposit in the glomeruli, resulting in proteinuria and reduced eGFR. Telitacicept simultaneously neutralizes BAFF and APRIL, suppressing pathogenic B-cell and plasma-cell responses, thereby reducing the levels of Gd-IgA1 (antigen) and autoantibodies while improving renal outcomes. Unlike conventional immunosuppressive agents that may disrupt the hypothalamic–pituitary–ovarian axis and impair fertility, selective BAFF/APRIL inhibition by telitacicept may preserve ovarian function and hormonal stability, thus supporting fertility preservation in women who may later wish to plan a pregnancy. Created with biogdp.com.

Telitacicept functions as a TACI-Fc fusion protein—integrating the transmembrane activator and calcium modulator and cyclophilin ligand interactor—to concurrently inhibit the activity of dual B-cell survival ligands, specifically BAFF and APRIL (Wu et al., 2023; Li S. et al., 2025). By competitively sequestering BAFF and APRIL, telitacicept prevents their binding to TACI, B-cell maturation antigen (BCMA), and B-cell activating factor receptor (BAFF-R). This blockade effectively suppresses the entire B-lymphocyte trajectory, from initial maturation to the final conversion into plasma cells that secrete antibodies (Zhang and Zhang, 2024; Shen et al., 2025). By inhibiting both signaling axes, telitacicept successfully lowers the concentration of disease-driving molecules, specifically autoantibodies (such as anti-dsDNA) in SLE and the pathogenic antigen (Gd-IgA1) in IgA nephropathy. Crucially, it maintains a degree of normal immune defense, which underpins its superior tolerability and safety (Cai et al., 2023). In IgAN, chronic overexpression of BAFF and APRIL is a primary driver for dysregulated B-cell responses, leading to the disproportionate synthesis of Gd-IgA1 (Zhuang et al., 2024). The accumulation of immune aggregates formed by Gd-IgA1 and its corresponding autoantibodies within the glomerular mesangium triggers activation and mesangial hypercellularity, ultimately advancing structural damage to the glomerulus (Zan et al., 2024). By concurrently neutralizing BAFF and APRIL, telitacicept markedly diminishes systemic Gd-IgA1 concentrations and IgA-based immune aggregates. This intervention alleviates mesangial inflammation and reduces immune-complex lodging by targeting the fundamental pathological origins of the disease (Cai et al., 2023; Wu et al., 2023; Liang and Li, 2026). The significance of the BAFF/APRIL signaling pathway in IgAN is further validated by genomic data. Evidence from Mendelian randomization indicates that aberrant B-cell activity and impaired IgA regulation constitute central pathogenic intersections, shaped by the interplay of genetic predispositions and external triggers. The immediate disruption of this integrated pathway by telitacicept translates into a unique therapeutic edge through its precision-targeted action on IgAN pathogenesis (Li C. et al., 2025). The intra-glomerular accrual of disease-causing autoantibodies and immune aggregates in SLE/LN initiates complement-mediated responses and podocyte dysfunction. These events fuel cascading inflammatory processes that ultimately culminate in progressive renal impairment (Zhang and Zhang, 2024). Chronic elevations of BAFF and APRIL in SLE and LN populations sustain the life cycle of self-reactive B cells and drive their maturation into plasma cells. This pathological signaling intensifies the secretion of anti-dsDNA antibodies, thereby escalating immune complex accumulation and the subsequent depletion of complement components (Fan et al., 2022). By targeting the BAFF/APRIL–TACI interaction, telitacicept effectively inhibits pathological B-lymphocyte activity at its source. This leads to a marked decline in systemic IgG, IgA, and IgM concentrations, along with specific autoantibodies, which, in turn, halts complement depletion and mitigates histological damage driven by immune aggregates. The therapeutic impact of this dual-target approach is validated by data showing that patients receiving telitacicept achieve substantial reductions in both clinical activity markers and protein excretion, along with recovery of complement components C3 and C4 (Wu et al., 2024). Furthermore, data from prospective longitudinal analyses confirm that telitacicept maintains long-term effectiveness and remains well tolerated over extended periods of immune modulation (Chen et al., 2025). The mechanistic pathways underlying these effects are summarized in Figure 1 (Jiang et al., 2025).

### 4.2 Real-world efficacy and safety evidence of telitacicept in IgAN and SLE/LN

#### 4.2.1 IgA nephropathy: deep remission and stabilization of renal function

In the therapeutic evaluation of IgAN, a phase II randomized controlled trial (RCT) conducted by Lv et al. provided evidence supporting the clinical efficacy of telitacicept. Clinical findings revealed that a 24-week regimen of 240 mg telitacicept yielded a 49.1% average decline in 24-h urinary protein (24 h-UP) levels relative to baseline. This therapeutic outcome markedly surpassed the minimal changes recorded in the placebo cohort. Importantly, the therapeutic benefits observed remained remarkably durable even after treatment cessation, as evidenced by clinical data during the post-intervention monitoring phase. Mechanistic analyses further revealed dose-dependent reductions in serum immunoglobulin A (IgA), immunoglobulin G (IgG), and immunoglobulin M (IgM) levels, indicating effective suppression of immune complex deposition within the glomeruli (Lv et al., 2023). For female patients with reproductive plans, achievement of deep proteinuria remission and targeted modulation of disease-driving mechanisms may substantially reduce pregnancy-associated renal hemodynamic stress, thereby providing a critical physiological foundation for safer conception. The concordant decline in proteinuria and total immunoglobulin levels supports the notion that telitacicept improves disease activity by inhibiting aberrant IgA production and reducing immune complex burden. Parallel therapeutic success has been documented in IgAN patients using a 240 mg weekly dose of telitacicept.

This regimen led to a near-halving of 24 h-UP levels within 24 weeks, while eGFR remained either stable or slightly elevated, demonstrating no meaningful renal decline (Wu et al., 2023). As highlighted by Liu et al., telitacicept, when added to therapy, enhances clinical control and serves as a protective alternative to traditional agents, such as MMF and CYC. This is particularly valuable to reduce the cumulative burden of drugs known for significant gonadal toxicity (Liu Y. et al., 2024). Empirical data from clinical practice further validate these outcomes, showing that eGFR levels remain largely unchanged after initiating telitacicept. Significantly, the lack of any clinical decline relative to pre-treatment states was paired with evidence of slight renal recovery in certain patient clusters (Weng et al., 2025).

#### 4.2.2 Systemic lupus erythematosus and lupus nephritis: disease control and immune reconstitution

As a dual BAFF/APRIL inhibitor, telitacicept has demonstrated clinical advantages in both induction and maintenance therapy for active SLE. A phase IIb clinical trial by Wu et al. provided compelling evidence of its efficacy: at week 48, the systemic lupus erythematosus responder index–4 (SRI-4) response rate in the telitacicept 240 mg group reached 75.8%, compared with 33.9% in the placebo group (*P* < 0.001) (Wu et al., 2024). Notably, the authors confirmed a pronounced steroid-sparing effect, with approximately 40.7% of patients successfully reducing their prednisone dose to ≤7.5 mg/day, nearly twice the proportion observed in the control group. The significant steroid-sparing potential of this therapy aligns with the fundamental priorities for female patients of childbearing age, particularly in safeguarding the integrity of the HPG axis and mitigating chronic metabolic side effects (Wu et al., 2024). These observations are further supported by Chan et al., who conducted a systematic review of randomized trials and found that telitacicept improves clinical outcomes across major composite assessments, including both the SRI-4 and BILAG-based combined lupus assessment (BICLA) scoring systems. When integrated with conventional care, telitacicept’s therapeutic impact matches that of established biologics like belimumab while offering advantages in specific clinical metrics, establishing it as a potent alternative for sustained management of SLE (Chan et al., 2023). Recent retrospective studies and real-world data have further supported the clinical efficacy of telitacicept in treating LN. Hu et al. demonstrated its capacity to induce deep remission in LN, supported by improvements in both renal and serological parameters. During follow-up, median 24 h-UP decreased rapidly from a baseline of 4.0 to 0.83 g/day (*P* < 0.001), while anti-dsDNA antibody titers declined markedly from 120 to 13 IU/mL. Concurrently, complement reconstitution was observed, with Complement component 3 (C3) levels increasing from 0.56 to 0.84 g/L, and Complement component 4 (C4) levels from 0.1 to 0.22 g/L (*P* < 0.001). The synchronized recovery of complement levels, along with the clearance of pathogenic autoantibodies, demonstrates that immune complex formation and renal deposition were successfully inhibited. This suppression allows high-risk LN patients to reach immune dormancy and stabilize kidney function before conceiving (Hu et al., 2025). Such concurrent normalization of serological and renal indicators is especially significant for high-risk LN cohorts, echoing the clinical management strategies for vulnerable populations established by Ku et al. (2016). The durable disease stability and low relapse rates associated with telitacicept therapy, therefore, provide a strong clinical foundation for women of childbearing age to achieve optimal disease control before conception. From a renal outcome perspective, telitacicept has consistently demonstrated favorable effects. Consistent with its role in modulating B-cell activity, therapy consistently led to marked decreases in serum IgG, IgM, and IgA, while simultaneously normalizing complement concentrations across various clinical trials (Wu et al., 2024; Liu et al., 2025). Telitacicept-treated cohorts have demonstrated superior 52-week complete renal response (CRR) and overall response rates (ORR). Moreover, propensity score-matched real-world evidence (RWE) indicate that integrating telitacicept into standard therapy markedly increases the likelihood of achieving renal remission (Jin et al., 2025). Furthermore, the urine protein-to-creatinine ratio (UPCR) showed a more immediate and pronounced decline, suggesting a more rapid stabilization of proteinuria and a reduction in intrarenal inflammatory processes (Weng et al., 2025). This perspective is further corroborated by documented case reports. For example, Liu et al. (2023) described a patient with refractory LN unresponsive to conventional immunosuppressive therapy who achieved simultaneous renal and serological remission following treatment with telitacicept in combination with low-dose glucocorticoids, underscoring its potential value in complex and treatment-resistant cases. Regarding dose optimization, a meta-analysis by Gao et al. showed no significant difference between standard-dose (160 mg/week) and higher-dose regimens in achieving primary efficacy endpoints, such as SRI-4, and no meaningful increase in adverse events. This indicates that telitacicept at a standard dosage effectively achieves therapeutic targets and profound remission, thereby optimizing the balance between efficacy and limited drug exposure (Gao et al., 2025).

## 5 Analysis of fertility preservation with telitacicept-based therapy

### 5.1 Reduction of conventional drug exposure and the potential for female fertility preservation

In conventional SLE management, the extended use of glucocorticoids at moderate or high doses often compromises fertility by inducing chronic negative feedback on the HPG axis. Such interference frequently manifests as ovulatory failure, menstrual cycle disruptions, and systemic reproductive endocrine imbalances in younger female patients (Khizroeva et al., 2019). A growing body of clinical research and systematic meta-analyses suggests that biological agents, by virtue of their precise immune-targeting properties, facilitate a swifter reduction in corticosteroid dependence. This transition ensures sustained disease stability and delivers a substantial ‘steroid-sparing’ benefit to the patient. Such a benefit represents a key indirect pathway through which biological agents alleviate the suppressive effects of corticosteroids on ovarian function and reduce chronic metabolic risks (Guan et al., 2023). By targeting both BAFF and APRIL, telitacicept effectively facilitates a transition to minimal glucocorticoid maintenance (≤7.5 mg/day of prednisone) for a larger patient population, while simultaneously lowering the risk of clinical flares. Lowering the total burden of corticosteroids can, consequently, diminish the incidence of permanent organ impairment and cardiovascular complications typically triggered by prolonged high-dose glucocorticoid administration (Jin et al., 2025). Consequently, therapeutic regimens based on telitacicept facilitate long-term clinical stability while tapering the use of steroids and cytotoxic agents, which limits the risk of medication-induced impairment to female fertility. Substantial clinical data validate this approach. Evidence from a 2025 retrospective analysis of patients with non-severe lupus nephritis highlights telitacicept’s capacity to markedly lower daily glucocorticoid requirements throughout the maintenance period, as reported by Liu et al. No novel severe safety concerns were identified during the one-year observation period, further validating the favorable tolerability profile of telitacicept for extended clinical use (Liu et al., 2025). Consistent with these observations, Chan et al. (2023) emphasized the substantial reduction in corticosteroid burden facilitated by telitacicept. This benefit is especially vital for female patients of childbearing age, as it minimizes the detrimental impact of glucocorticoids on ovarian follicles and systemic hormonal balance, a conclusion reinforced by real-world evidence. Zhang S. et al. (2025) reported a case of IgAN in which the administration of telitacicept alongside a tapered glucocorticoid regimen resulted in a significant decline in urinary protein excretion and the restoration of renal stability, indicating that therapeutic success remains attainable even with a reduced corticosteroid burden.

Furthermore, evidence from a one-year real-world observational study indicates that the durable response to telitacicept enables patients to maintain low disease activity while markedly tapering their reliance on intensive glucocorticoid therapy (Jin et al., 2025). Such a safety profile underscores telitacicept’s potential as a more viable and enduring treatment strategy for female patients of childbearing age suffering from SLE and IgAN (Hu et al., 2025).

### 5.2 Ovarian protection and female fertility preservation with telitacicept

Conventional cytotoxic immunosuppressive therapies are associated with substantial risks of ovarian toxicity in women of reproductive age with SLE. A prospective study by Sharma et al. (2020) demonstrated that even moderate-dose intravenous CYC induction therapy administered for 6 months resulted in a significant decline in ovarian reserve. Precisely, a marked decline in serum AMH and inhibin B was observed alongside an elevation in follicle-stimulating hormone (FSH) concentrations, reflecting a malfunction in the regulatory feedback of the hypothalamic-pituitary-ovarian (HPO) axis. Ultrasound imaging additionally detected a decline in ovarian size and a lower number of antral follicles. Although menstrual cycles resumed in some patients during follow-up, the combined clinical and subclinical findings indicated persistent adverse effects on female fertility associated with moderate-dose CYC exposure.

In contrast, prospective clinical data suggest that telitacicept has a favorable ovarian safety profile. A prospective cohort study by Zhang Z. et al. (2025) involving adult female patients with SLE demonstrated that ovarian reserve markers, including AMH and gonadal axis hormones, such as FSH and luteinizing hormone (LH), remained stable over a 6-month telitacicept treatment period. Estradiol (E2) levels also remained within physiological ranges; no deterioration in ovarian reserve was observed. Furthermore, telitacicept treatment was not associated with an increased incidence of menstrual cycle irregularities or amenorrhea, indicating preservation of HPO axis rhythmicity. Unlike conventional cytotoxic immunosuppressants, telitacicept provides effective immunomodulation without compromising ovarian reserve or endocrine stability.

### 5.3 Clinical evidence supporting improved pregnancy outcomes

Telitacicept provides distinct therapeutic gains in SLE and IgAN by balancing durable disease control with kidney preservation. By reducing exposure to agents with known risks to female fertility, this approach creates a robust medical rationale for optimizing fertility outcomes and successful pregnancies (Ku et al., 2016; Wind et al., 2024). Lian et al. (2021) reported that individuals with established lupus nephritis prior to conception face a greater risk of combined adverse fetal complications (CAFOs) than those whose renal involvement first manifests during the gestational period, underscoring the importance of achieving deep and sustained remission before conception. Similarly, Fang et al. (2023) demonstrated that maternal–fetal outcomes are significantly better in women who conceive during stable chronic kidney disease (CKD) stages, particularly CKD 1–2, than in those with renal deterioration during pregnancy. Together, these findings consistently support low disease activity and stable renal function prior to conception as prerequisites for safe pregnancy in women with SLE/LN and IgAN. By simultaneously inhibiting the BAFF/APRIL signaling axis, telitacicept stabilizes complement levels and reduces anti-dsDNA concentrations. This multi-target action extends remission duration and helps maintain minimal disease activity before conception (Zhu et al., 2024; Liang and Li, 2026). Furthermore, results from cohort analyses indicate that high SLEDAI scores prior to pregnancy and low complement levels are independent risk factors for both AMOs and adverse neonatal outcomes (ANOs), underscoring that maintaining immunological equilibrium is fundamental to successful gestational counseling (Lu et al., 2024). A comparable clinical framework is gaining recognition within the management of IgAN. A case of a healthy gestation following telitacicept administration was reported in the clinical findings of Du et al. (2024), suggesting that durable remission can be maintained upon discontinuation of teratogenic agents such as MMF. Renal function remains a key determinant of pregnancy risk: Shimizu et al. (2015) demonstrated that women with preconception eGFR ≥ 45 mL/min did not experience significant postpartum renal decline, whereas those with eGFR < 45 mL/min showed accelerated deterioration. Consistent with these findings, extensive cohort data have shown that advanced CKD (eGFR below 60 mL/min/1.73 m^2^) is a standalone predictor of poor gestational outcomes and a permanent decline in kidney function in patients with IgAN (Su et al., 2017). Since pregnancy-related physiological changes often exacerbate protein leakage in late gestation and the puerperium, pre-pregnancy protein control becomes a vital strategy for safeguarding the health of both mother and child. Owing to its capacity to rapidly control protein excretion and preserve eGFR, telitacicept offers significant potential to refine the pre-pregnancy renal status of female patients with IgAN (Zhang S. et al., 2025). Evidence from both controlled trials and observational studies provides reassurance, with no increased risk of severe infectious diseases or impairment to female fertility (Liu L. et al., 2024).

### 5.4 Real-world pregnancy case: from deep remission to successful delivery

Du et al. (2024) reported the first documented case of a patient with refractory IgAN who successfully conceived and delivered a healthy infant following telitacicept therapy. The patient had shown inadequate responses to conventional immunosuppressive treatments; however, after initiation of telitacicept, 24 h-UP rapidly declined to remission levels (<0.5 g/24 h), accompanied by marked improvement in renal function.

Importantly, the patient conceived naturally 3 months after discontinuation of telitacicept, and disease activity remained quiescent throughout pregnancy, with no relapse or pregnancy-related complications. This observation reinforces the utility of telitacicept as a preliminary intervention to induce remission, demonstrating its ability to provide high-risk women with a reliable opportunity for conception by normalizing immune responses. The clinical significance and key mechanisms of telitacicept in renal protection, steroid-sparing, and ovarian function preservation are summarized in Table 2.

**TABLE 2:** Fertility preservation and clinical considerations of telitacicept in women of childbearing age with SLE or IgA nephropathy: mechanisms and evidence.

Domain	Key mechanisms/Evidence	Clinical significance
Immunological mechanisms	1. Dual targeting of BAFF/April inhibits autoreactive B cells and plasma cell generation 2. Lowers pathogenic Gd-IgA1 (antigen), IgA-ICs, and SLE-related autoantibodies (anti-dsDNA) 3. Elevates C3/C4, improves complement consumption status	1. Controls immune dysregulation at the source, reduces inflammatory load 2. Provides possibility of achieving stable remission pre-conception 3. Reduces risk of disease flare during pregnancy
Renal protection and pre-conception optimization	1. IgAN: Significant reduction in proteinuria at 24 weeks, stable eGFR 2. RWE shows long-term eGFR non-inferiority, improvement in some patients 3. SLE/LN: Rapid decline in proteinuria, improved CRR/ORR	1. Reduces pre-conception proteinuria load (IgAN is a key risk factor) 2. Maintains stable renal function, raises pregnancy safety threshold (esp. CKD 1–2) 3. Avoids risk of post-pregnancy renal deterioration in eGFR <45 mL/min population
Reducing traditional drug exposure (steroid sparing + reduced toxicity)	1. Increases proportion achieving low-dose GC (≤7.5 mg/d) 2. Lowers demand for CYC/MMF, avoiding cytotoxicity and teratogenic risks 3. RWE confirms significant steroid dose reduction at 12 months	1. Avoids HPG axis suppression (steroids) 2. Reduces risk of amenorrhea/ovarian reserve decline (CYC) 3. Avoids the teratogenic risk associated with MMF→ provides more sustainable pre-conception maintenance plan
Ovarian function and reproductive-endocrine safety	1. 6 months telitacicept: Stable AMH, FSH, LH, E2 levels 2. No observed decrease in ovarian reserve or increase in menstrual disorders	1. Does not damage ovarian reserve (vs. Significant toxicity of CYC) 2. Does not interfere with HPG axis, beneficial for maintaining ovulation 3. Suitable for reproductive-age women requiring long-term maintenance
Pregnancy and fetal prognosis	1. IgAN: First reported successful pregnancy case post-Telitacicept 2. Improves pre-conception baseline by reducing proteinuria and stabilizing eGFR 3. Offers possibility of pre-conception complete/deep remission (preventing LN flare)	1. Helps obtain better pre-conception disease control — the core premise for good pregnancy prognosis 2. Reduces risks of preterm birth, fetal growth restriction, APOs 3.Provides safer long-term management strategy for high-risk SLE/IgAN pregnancies
Overall clinical value	1. Good safety profile: No increase in serious infections or adverse effects on gonadal function and fertility 2. No need for blood concentration monitoring, simpler management than TAC	1. More suitable for long-term use before planned pregnancy 2. Improves long-term compliance 3. More suitable as a cornerstone for maintenance therapy during the “pre-conception optimization phase”

## 6 Conclusion and future perspectives

Dual inhibition of BAFF and APRIL represents a pharmacologically rational strategy for immune-mediated renal diseases driven by pathogenic B-cell activation. Through targeted suppression of autoreactive B cells and plasma cells, telitacicept achieves effective control of antibody-mediated inflammation without relying on cytotoxic or DNA-damaging mechanisms. Clinical trials and real-world evidence demonstrate that telitacicept reduces disease activity, proteinuria, and pathogenic serological markers in systemic lupus erythematosus, lupus nephritis, and refractory IgA nephropathy, while providing consistent steroid-sparing effects and favorable tolerability. This profile supports its potential role in long-term maintenance therapy, an area of persistent unmet need. Telitacicept demonstrates significant potential for female fertility preservation by safeguarding ovarian reserve markers and mitigating gonadotoxicity. However, its safety during pregnancy and lactation remains a critical knowledge gap; specifically, data on transplacental passage and breast milk excretion are currently unavailable. This evidence gap complicates clinical counseling. Clinicians and patients must therefore navigate treatment decisions without definitive safety benchmarks. Future prospective studies are essential to evaluate gestational outcomes and bridge the gap between effective disease control and fertility preservation.

## Data Availability

The original contributions presented in the study are included in the article/supplementary material, further inquiries can be directed to the corresponding author.

## Glossary

SLE: Systemic lupus erythematosus
IgAN: IgA nephropathy
Gd-IgA1: Galactose-deficient IgA1
TACI: Transmembrane Activator and CAML Interactor
Fc: Fragment crystallizable
BAFF: B-cell activating factor
APRIL: A Proliferation-inducing ligand
APOs: Adverse pregnancy outcomes
LN: Lupus nephritis
CYC: Cyclophosphamide
MMF: Mycophenolate Mofetil
HPG: Hypothalamic–pituitary–gonadal
eGFR: Glomerular filtration rate
APS: Antiphospholipid syndrome
aPL: Antiphospholipid antibodies
SLEDAI: Systemic Lupus Erythematosus Disease Activity Index
AMH: Anti-Müllerian hormone
TAC: Tacrolimus
AMOs: Adverse maternal outcomes
CNI: Calcineurin inhibitor
TDM: Therapeutic Drug Monitoring
BCMA: B-cell maturation antigen
BAFF-R: B-cell activating factor receptor
anti-dsDNA: Anti-double-stranded DNA antibodies
RCT: Randomized controlled trial
24 h-UP: 24-h urinary protein
IgA: Immunoglobulin A
IgG: Immunoglobulin G
IgM: Immunoglobulin M
SRI-4: Systemic Lupus Erythematosus Responder Index–4
BICLA: BILAG-based Combined Lupus Assessment
C3: Complement component 3
C4: Complement component 4
CRR: Complete renal response
ORR: Overall response rates
RWE: Real-World Evidence
UPCR: Urine protein-to-creatinine ratio
FSH: Follicle-stimulating hormone
HPO: Hypothalamic-Pituitary-Ovarian
LH: Luteinizing hormone
E2: Estradiol
AFOs: Adverse fetal complications
CKD: Chronic kidney disease
ANOs: Adverse Neonatal Outcomes
